# Co-production of farnesol and coenzyme Q_10_ from metabolically engineered *Rhodobacter sphaeroides*

**DOI:** 10.1186/s12934-019-1145-6

**Published:** 2019-05-31

**Authors:** Xueduan Chen, Xianzhang Jiang, Man Xu, Mingliang Zhang, Runye Huang, Jianzhong Huang, Feng Qi

**Affiliations:** 1 Engineering Research Center of Industrial Microbiology of Ministry of Education, College of Life Sciences, Fujian Normal University, Fuzhou, 350117 Fujian China; 2 Provincial University Key Laboratory of Cellular Stress Response and Metabolic Regulation & Fujian Provincial University Engineering Research Center of Industrial Biocatalysis, Fujian Normal University, Fuzhou, 350117 Fujian China

**Keywords:** Farnesol, CoQ_10_, PSY, RNAi, Carbon flux

## Abstract

**Background:**

Farnesol is an acyclic sesquiterpene alcohol present in the essential oils of various plants in nature. It has been reported to be valuable in medical applications, such as alleviation of allergic asthma, gliosis, and edema as well as anti-cancerous and anti-inflammatory effects. Coenzyme Q_10_ (CoQ_10_), an essential cofactor in the aerobic respiratory electron transport chain, has attracted growing interest owing to its clinical benefits and important applications in the pharmaceutical, food, and health industries. In this work, co-production of (*E*,*E*)-farnesol (FOH) and CoQ_10_ was achieved by combining 3 different exogenous terpenes or sesquiterpene synthase with the RNA interference of *psy* (responsible for phytoene synthesis in *Rhodobacter sphaeroides* GY-2).

**Results:**

FOH production was significantly increased by overexpressing exogenous terpene synthase (TPS), phosphatidylglycerophosphatase B (PgpB), and sesquiterpene synthase (ATPS), as well as RNAi-mediated silencing of *psy* coding phytoene synthase (PSY) in *R. sphaeroides* strains. Rs-TPS, Rs-ATPS, and Rs-PgpB respectively produced 68.2%, 43.4%, and 21.9% higher FOH titers than that of the control strain. Interestingly, the CoQ_10_ production of these 3 recombinant *R. sphaeroides* strains was exactly opposite to that of FOH. However, CoQ_10_ production was almost unaffected in *R. sphaeroides* strains modified by *psy* RNA interference. The highest FOH production of 40.45 mg/L, which was twice as high as that of the control, was obtained from the TPS-PSYi strain, where the exogenous TPS was combined with the weakening of the phytoene synthesis pathway via *psy* RNA interference. CoQ_10_ production in TPS-PSYi, ATPS-PSYi, and PgpB-PSYi was decreased and lower than that of the control strain.

**Conclusions:**

The original flux that contributed to phytoene synthesis was effectively redirected to provide precursors toward FOH or CoQ_10_ synthesis via *psy* RNA interference, which led to weakened carotenoid synthesis. The improved flux that was originally involved in CoQ_10_ production and phytoene synthesis was redirected toward FOH synthesis via metabolic modification. This is the first reported instance of FOH and CoQ_10_ co-production in *R. sphaeroides* using a metabolic engineering strategy.

**Electronic supplementary material:**

The online version of this article (10.1186/s12934-019-1145-6) contains supplementary material, which is available to authorized users.

## Background

**Fig. 1:**
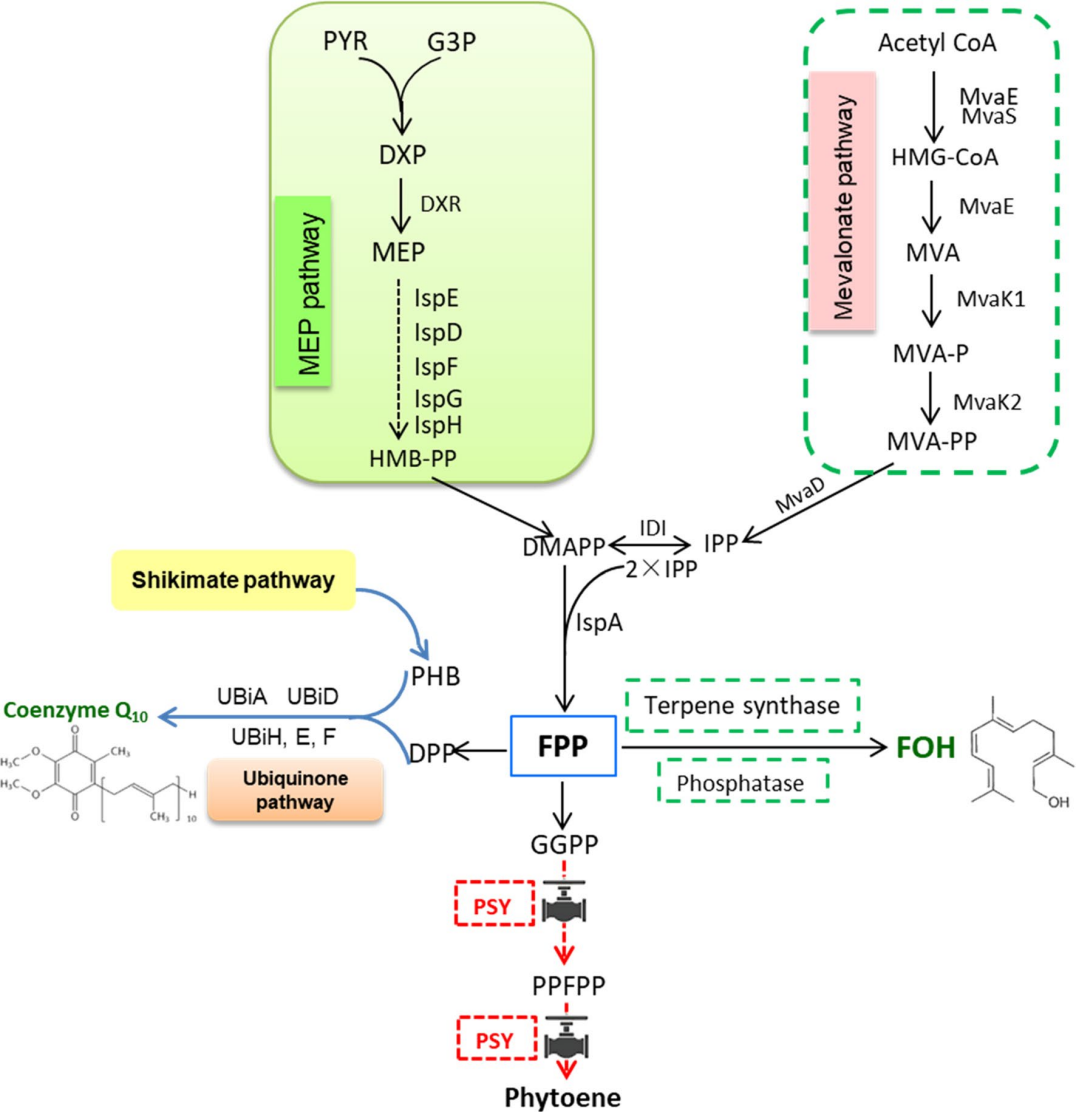
Schematic presentation of the strategy for improved co-production of FOH and CoQ_10_, including introduction of three different exogenous terpene synthase or phosphatase combined with RNAi mediated silencing of gene *psy* of phytoene synthesis pathway in *R. sphaeroides* GY-2

Sesquiterpenes are C15-terpenoids composed of 3 isoprene units with numerous important medical and industrial applications [1]. (*E*,*E*)-Farnesol (FOH; C_15_H_26_O) is a colorless acyclic sesquiterpene alcohol found in many essential plant oils such as lemon grass, neroli, cyclamen, tuberose, rose, and musk [2]. FOH is known to play an important role in signal transduction [3], cell proliferation, quorum-sensing [4, 5], and apoptosis induction [6, 7]. Recently, FOH was reported to be valuable in medical application because it exhibited anti-cancerous and anti-inflammatory effects, and was also found to alleviate allergic asthma, gliosis, and edema [8, 9]. However, FOH extraction from plants is non-feasible for large scale production as only trace amounts can be found in plant essential oils. Furthermore, the plant resources for FOH production are limited to slow growth, varying composition, season, climate, and scaling-up facilitation. In order to circumvent these limitations, FOH production in prokaryotic and eukaryotic microorganisms using metabolic engineering has been exploited [10–13], along with culture optimization for enhanced FOH production using *Saccharomyces cerevisiae* and *Candida albicans* [14, 15]. Figure 1 illustrates that FOH can be obtained from the depyrophosphorylation of farnesyl pyrophosphate (FPP) catalyzed by phosphatases or sesquiterpene synthases [11]. Unfortunately, only a few studies discuss FOH production by metabolically engineering microbial hosts, even though novel medical and industrial advantages of FOH have been revealed.

The ubiquinone, coenzyme Q_10_ (CoQ_10_; C_59_H_90_O_4_), is an essential cofactor in the aerobic respiratory electron transport chain and is widely distributed in almost all organisms [16, 17]. CoQ_10_ is composed of a benzoquinone head group and a side chain composed of 10 isoprenoid units, which distinguishes it from other CoQ_n_ cognates. CoQ_10_ is embedded within the hydrophobic domain of the phospholipid bilayer of cytoplasmic membranes, and plays an essential role in removing harmful reactive oxygen species as a redox-active molecule during disulfide bond formation [18, 19]. The clinical benefits of CoQ_10_ supplementation include the prevention of heart failure as a supplement to 3-hydroxy-3-methylglutaryl coenzyme A (HMG-CoA) reductase inhibitors [20, 21] and acute myocardial infarction in cardiovascular diseases, as well as positive effects in mitochondrial diseases, certain neurodegenerative diseases, and diabetes [22, 23]. Over the past 2 decades, economical production of CoQ_10_ using microbial processes has been developed because of the growing demands for CoQ_10_ in the pharmaceutical and food industry. Some prokaryotic microorganisms, such as *Rhodobacter sphaeroides* and *Agrobacterium tumefaciens*, have been well-studied by researchers as natural producers of CoQ_10_ [20, 24, 25]. In most prokaryotic cells, there are 3 combined pathways involved in CoQ_10_ biosynthesis. The CoQ_10_ benzoquinone “head” is synthesized from 4-hydroxybenzoic acid via the shikimate pathway, whereas the isoprenoid side chains are synthesized via isopentenyl pyrophosphate (IPP) synthesis enzymes using 10 IPP molecules derived from pyruvate and glyceraldehyde-3-phosphate from the methylerythritol phosphate (MEP) or mevalonate (MVA) pathways that are only present in eukaryotes and some bacteria. CoQ_10_ is then assembled by combining the isoprenoid side chain with the benzoquinone head in the ubiquinone pathway [17, 18] (Fig. 1).

**Fig. 2:**
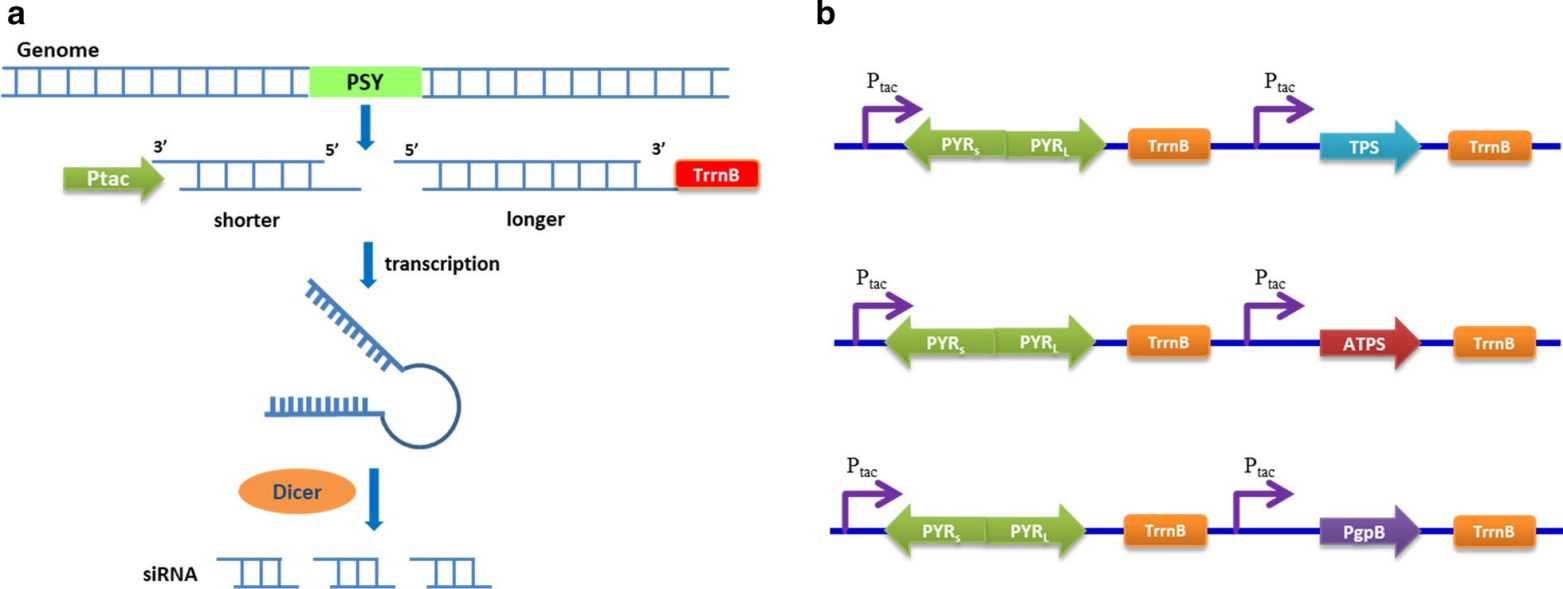
Schematic diagram of the methods used in this work for redirection of carbon flux. **a** The inducible gene silencing was triggered by expressing a hairpin RNA. After design and induction of the hairpin dsRNA, the dsRNA can be digested by the dicer enzyme in the cell to produce small interference RNA (siRNA) that triggers RNA interference (RNAi) to specifically degrade the endogenous target mRNA. **b** The short and long fragments of *psy* are constructed for conformation of the hairpin dsRNA under the control of tac promoter, as well as the exogenous genes coding TPS, PgpB and ATPS

The synthesized FPP from IPP and dimethylallyl pyrophosphate (DMAPP) is distributed into different pathways, including lycopene, ubiquinone, or squalene biosynthesis, in prokaryotic microorganisms. *R. sphaeroides* is utilized for industrial CoQ_10_ production because a large proportion of FPP can be directed to the ubiquinone synthesis pathway for high-titer CoQ_10_ production, in addition to the detailed knowledge of the key genes and metabolic pathways involved in CoQ_10_ biosynthesis [17, 26]. The excess FPP can be used as a precursor for the synthesis of other valuable compounds. In this study, *R. sphaeroides* GY-2, a high-yield CoQ_10_ mutant strain obtained from *R. sphaeroides* 2.4.1 (ATCC 17023) in our previous work [26], was metabolically engineered to directly produce FOH by introducing sesquiterpene synthase or terpene synthase. In order to improve the FPP supplement for FOH biosynthesis, the competitive phytoene synthesis pathway was weakened by efficiently downregulating the *psy*-coding phytoene synthase (PSY) using RNAi-mediated gene silencing (Fig. 2a). Furthermore, the exogenous genes of phosphatidylglycerophosphatase B (PgpB, GeneID: 945863) from *Escherichia coli*, terpene synthase (TPS, GeneID: 541974) from *Zea mays*, and acyclic sesquiterpene synthase (ATPS, GeneID: 100833760) from *Brachypodium distachyon* were introduced into *R. sphaeroides* GY-2 for FOH synthesis (Fig. 2b). FOH is synthesized in the cytoplasm and secreted outside the cells, whereas the produced CoQ_10_ is maintained inside cells and attached to the cell membrane. Therefore, FOH and CoQ_10_ can theoretically be co-produced simultaneously via separation of supernatant and cell pellet. To the best of our knowledge, this is the first report that is looking to synthesize FOH in *R. sphaeroides* while attempting to co-produce FOH and CoQ_10_ at the same time.

## Materials and methods

### Chemicals and regents

All the restriction enzymes in this study were purchased from New England Biolabs (Beijing, China). The gel extraction kits and plasmid mini kits were from Omega bio-tek (Guangzhou, China). The standard of farnesol and *n*-decane are from Sigma-Aldrich Inc., (Milwaukee, Wisconsin, US). The fermentation medium and chemicals were purchased from Beijing Chemical Works (Beijing, China).

### Strains and culture conditions

*Escherichia coli* DH5α and *E. coli* TOP10 were used for cloning and plasmids construction. *E. coli* S17-1 is the plasmid donor cells that transport recombinant plasmids into *R. sphaeroides* strains for bi-parental conjugation. *R. sphaeroides* GY-2 that is a mutant strain with high yield of CoQ_10_ was derived from *R. sphaeroides* 2.4.1. The strains and plasmids used in this study are listed in Table 1. *E. coli* strains were cultivated at 37 °C in Luria–Bertani (LB) medium supplemented with 50 mg/mL of kanamycin sulfate when necessary. *R. sphaeroides* strains were grown at 32 °C in the seed culture medium composed of 3.5 g/L (NH_4_)_2_SO_4_, 2 g/L yeast extract, 0.75 g/L corn steep powder, 10 g/L glucose, 2 g/L NaCl, 0.75 g/L K_2_HPO_4_, 0.75 g/L KH_2_PO_4_, 0.1 g/L FeSO_4_ and 0.3 g/L MgSO_4_, 1 g/L nicotinic acid (Amresco Ltd., Solon, US), and 1 g/L biotin (Sigma-Aldrich, St Louis, US) at the pH 6.1–6.3 adjusted by 10 mol/L sodium hydroxide. The fermentation culture medium was composed of 4 g/L (NH_4_)_2_SO_4_, 32.5 g/L glucose, 3.25 g/L NaCl, 1.2 g/L KH_2_PO_4_, 1.4 g/L FeSO_4_ and 9.8 g/L MgSO_4_, 3.9 g/L corn steep powder with an initial pH 6.0-6.3.

**Table 1:** Strains, plasmids and primers used in this study

	Description	References
Strain		
*E. coli* S17-1	RP4-2. Tc::Mu-Km::Tn7	[27]
*E. coli* DH5α	F-λ-endA1 glnV44 thi-1 recA1 relA1 gyrA96 deoR nupG Φ80dlacZΔM15 Δ(lacZYA-argF)U169, hsdR17(rK-mK+)	Invitrogen
*E. coli* TOP10	F-mcrA Δ(mrr-hsdRMS-mcrBC) φ80lacZΔM15 ΔlacX74 recA1 araD139	Invitrogen
	Δ(ara-leu)7697 galU galK λ-rpsL(StrR) endA1 nupG	Invitrogen
*R. sphaeroides* GY-2	CoQ10 high-yield mutant strain obtained from *R. sphaeroides* 2.4.1	This study
Rs-TPS	*R. sphaeroides* GY-2 harboring TPS	This study
Rs-ATPS	*R. sphaeroides* GY-2 harboring ATPS	This study
Rs-PgpB	*R. sphaeroides* GY-2 harboring PgpB	This study
PSYi1, PSYi2, PSYi3, PSYi4	*R. sphaeroides* GY-2 derivatives with *psy* RNA interference	This study
TPS-PSYi	*R. sphaeroides* GY-2 harboring TPS with *psy* RNA interference	This study
ATPS-PSYi	*R. sphaeroides* GY-2 harboring ATPS with *psy* RNA interference	This study
PgpB-PSYi	*R. sphaeroides* GY-2 harboring PgpB with *psy* RNA interference	This study
Plasmids		
pYC6a	Ptac promoter, AmpR	This study
pBBR1MCS2	low-copy cloning vector, KanR	[28]
pBBR1MCS2-tac	pBBR1MCS2 containing tac promoter	This study
pBBR1MCS2-PgpB	pBBR1MCS2-tac containing PgpB from *E. coli*	This study
pBBR1MCS2-ATPS	pBBR1MCS2-tac containing ATPS from *Brachypodium distachyon*	This study
pBBR1MCS2-TPS	pBBR1MCS2-tac containing TPS from *Zea mays*	This study
pBBR1MCS2-PSYi	pBBR1MCS2-tac containing PSYi	This study
pBBR1MCS2-PSYi-PgpB	pBBR1MCS2-PSYi containing PgpB from *E. coli*	This study
pBBR1MCS2-PSYi-ATPS	pBBR1MCS2-PSYi containing ATPS from *Brachypodium distachyon*	This study
pBBR1MCS2-PSYi-TPS	pBBR1MCS2-PSYi containing TPS from *Zea mays*	This study

Primers	Sequence (5′–3′)	
PTR-F	ATTCCCCGCGGCACAGCTAACACCACGTCGTC	
PTR-R	GAGCCCAAGCTTGAAAGGCCCAGTCTTTCGAC	
PSYLi-F	GGAATTCCGGCGGCGATGGCCGCGCCCC	
PSYLi-R	TTCCGCGGCCGCTATGGCCGACGTCGACGCCAGGCCGCGAGCCCCGCGG	
PSYSi-F	GGAATTCCCTCGAGGTGGCGCGCACGCC	
PSYSi-R	CGAGCTCGCCAGGCCGCGAGCCCCGC	
pBBR1MCS2-F	GAATTCGGTGAGCTCGGTCTG	
pBBR1MCS2-R	GGTTAATTCCTCCTGTTACGCGC	
PgpB-EcoF	CGTAACAGGAGGAATTAACCATGCGTTCGATTGCCAGAC	
PgpB-EcoR	AGACCGAGCTCACCGAATTCTTAGTGGTGGTGGTGGTGGTG	
ATPS-F	CGTAACAGGAGGAATTAACCATGCACATCGACCCGGCC	
ATPS-R	AGACCGAGCTCACCGAATTCTTAGAGCGAGTCCGAGAAGTCG	
TPS-F	CGTAACAGGAGGAATTAACCATGGCCATGCCGGTGAAG	
TPS-R	AGACCGAGCTCACCGAATTCTTACACGTCCGACACGAG	
PSYitac-F	CGCGGATCCCACAGCTAACACCACGTCG	
PSYiT-R	TTGGCGCGCCTAGTGGTGGTGGTGGTGGT	

### Construction of vectors

The expression plasmid pBBR1MCS2 was modified using pYC6a. The fragment containing the tac promoter and TrrnB terminator was amplified from pYC6a using primers PTR-F and PTR-R. The resulting fragment was digested using *Sac*II and *Hin*dIII, and ligated into pBBR1MCS2 to substitute the lac promoter of the original pBBR1MCS2 with tac promoter, resulting in the plasmid pBBR1MCS2-tac. The gene encoding PgpB from *E. coli* Top 10 was amplified using the primers PgpB-EcoF and PgpB-EcoR listed in Table 1, and ligated with PCR product of pBBR1MCS2 (with primers pBBR1MCS2-F and pBBR1MCS2-R) using Gibson Assembly master mix (New England Biolabs, Beijing, China). The genes encoding ATPS from *Brachypodium distachyon* and TPS from *Zea mays* were codon optimized for expression in *R. sphaeroides*, and synthesized by GenScript Ltd (Nanjing, China). Then codon optimized ATPS and TPS have been ligated into pBBR1MCS2-tac by Gibson Assembly cloning method using the primers ATPS-F, ATPS-R and TPS-F, TPS-R, respectively (Fig. 2b). The protocol of RNAi-mediated gene silencing was followed the previous report [29]. The short (365 bp) and long fragment (462 bp) of *psy* were amplified from the genome of *R. sphaeroides* using the primers of PSYSi-F, PSYSi-R and PSYLi-F, PSYLi-R, respectively. Then the short fragment was digested by *Eco*RI and *Sac*I, whereas the long fragment was digested by *Eco*RI and *Sal*I. Then ligation was performed by mixing the two digested PCR products (827 bp) with the *Sac*I/*Sal*I digested vector pBBR1MCS2-tac, resulting in the pBBR1MCS2-PSYi that contained the inverted repeat of *psy*. The genes of PgpB, ATPS and TPS with tac promoter and TrrnB were amplified using the primers PSYitac-F and PSYitac-R, and digested with *Bam*HI/*Asc*I. The three fragments were ligated into the *Bam*HI/*Asc*I digested pBBR1MCS2-PSYi, resulting in the pBBR1MCS2-PSYi-PgpB, pBBR1MCS2-PSYi-ATPS and pBBR1MCS2-PSYi-TPS, respectively (Fig. 2a). Each plasmid and primer sequence was listed in Table 1.

### Bi-parental conjugation for transformation

All the pBBR1MCS2-tac derivative expression plasmids were transformed into *E. coli* S17-1 via electroporation and the resulting strains were used as plasmid donors. The process of conjugation mating was modified and carried out as described in a previous study [30]. Firstly, the donor strain *E. coli* S17-1 and the recipient *R. sphaeroides* GY-2 were grown to mid-log phase, harvested by centrifugation (8000*g*, 6 min) and resuspended in fresh LB medium. Then *E. coli* S17-1 and *R. sphaeroides* GY-2 cell suspensions were mixed at the ratios of 1:6 and 1:10, respectively. Afterwards, the culture mixture of the two strains was harvested, transferred to LB agar plate, and incubated for 24–30 h at 32 °C. The resulting strains were harvested by washing with 0.1 mL pre-cooled Sistrom’s minimal medium [31] and spread on LB agar plates containing 200 mg/L K_2_TeO_3_ and 50 mg/mL kanamycin. The black kanamycin-resistant colonies were isolated, verified by PCR analysis, and utilized for further experiments. The recombinant *R. sphaeroides* GY-2 harboring pBBR1MCS2-tac was used as the control.

### FOH and CoQ_10_ production

All the recombinant *R. sphaeroides* strains were inoculated and grown in the seed culture medium at pH 6.4 and 32 °C, under constant orbital shaking at 200 rpm for 24 h, and then transferred to 500 mL baffled flasks containing 150 mL medium for batch fermentation at the same conditions for 48 h. The method of FOH collection was carried out as described in the previous study [10]. In order to avoid volatile loss of FOH, the fermentation medium was covered with 15% volume of decane to collect FOH. After the batch fermentation, the upper layer of decane phase was collected for FOH extraction. Then the remaining solution was centrifuged for 8 min at 12,000 rpm to collect cells for CoQ_10_ extraction. The cells were re-suspended using 150 mL PBS solution and 1 mL aliquot of the fermentation broth was harvested, combined 200 μL HCl solution (6 mol/L), and incubated at 65 °C for 20 min. Subsequently, 1 mL acetone and 100 μL of 30% hydrogen peroxide solution were added. After that the mixture was collected, filled with ethanol up to 10 mL, and incubated in an ultrasonic bath at 4 °C for 45 min. Pellets were harvested and centrifuged at 8000*g* for 10 min. The resulting supernatants were collected and examined for CoQ_10_ production.

### HPLC and GC–MS analysis

The yield of FOH was detected by GC–MS (Agilent Technologies 6890 N Gas Chromatograph combined with 5975C Mass Spectrometry with Triple-Axis Detector) in this study. Samples were injected at a split ratio of 1:5 and were separated using a 19091N-133I HP-INNOWAX chromatographic column (30 m × 0.25 mm × 0.25 μm) with helium at the flow rate of 1.0 mL/min as carrier gas. The initial temperature of the injector was 250 °C and the column oven is set to 60 °C. In the first step of vaporization, the heating rate was 10 °C/min until the temperature rises to 190 °C and held for 2 min. The heating rate in the second step was 20 °C/min to the final temperature of 250 °C and held 5 min. The standard sample of FOH was purchased from Sigma-Aldrich Inc., (Shanghai, China). CoQ_10_ was determined using an UltiMate TM 3000 HPLC system (Thermo-Fisher Scientific, Waltham, US) equipped with a 250 mm × 4.6 mm × 5 µm, C18-reversed phase column (Thermo-Fisher Scientific, Beijing, China) and a photo-diode array detector. Methanol/isopropyl alcohol at a ratio of 3:1 (v/v) was used as the mobile phase at a flow rate of 0.8 mL/min. CoQ_10_ can be detected at 275 nm and 254 nm and its concentrations were determined using a standard curve based on an HPLC-grade authentic CoQ_10_ standard (Sigma-Aldrich, Shanghai, China).

### Quantitative real-time RT-PCR analysis

In order to evaluate the effects of RNA interference of the *psy* gene, quantitative real-time RT-PCR was utilized for analysis of all the PSY RNAi strains. The RNA samples for quantitative real-time RT-PCR analysis were collected from cells grown under the same growth conditions. Total RNA was isolated from all the PSY RNAi *R. sphaeroides* strains and reversely transcribed using a two-step strategy with reverse transcriptase PCR kit (Qiagen, Shanghai, China). The RT-qPCR reaction was carried out in 25 μL reactions containing 12.5 μL of Universal SYBR Master (ROX), 2.0 μL of properly diluted cDNA from 20–30 ng/mL of cDNA for all genes, 0.5 μL of 50× ROX reference dye II (for error correction between wells), and 0.5 ml of each primer at 10 mM and 9 mL of sterile distilled water. GAPDH gene of *R. sphaeroides* was selected as control for normalizing expression of the samples. All real-time PCR reactions were performed on the LightCycler^®^ 96 (Roche Diagnostics, US). The fold change of the target cDNA was determined using the value of $2^{{ - \Delta \Delta {\text{C}}_{\text{t}} }} \left( { - \Delta \Delta {\text{C}}_{\text{t}} = \left[ {{\text{C}}_{{{\text{t}}({\text{target}})}} - {\text{C}}_{{{\text{t}}({\text{ref}})}} } \right]_{\text{sample}} - \left[ {{\text{C}}_{{{\text{t}}({\text{target}})}} - {\text{C}}_{{{\text{t}}({\text{ref}})}} } \right]_{\text{WT}} } \right)$, where Ct represents the threshold cycle [32]. All the values were determined through triplicate experiments and statistical significance was considered significant at *P *< 0.05.

## Results

### Introduction of exogenous phosphatases, terpene, and sesquiterpene synthase for FOH production

**Fig. 3:**
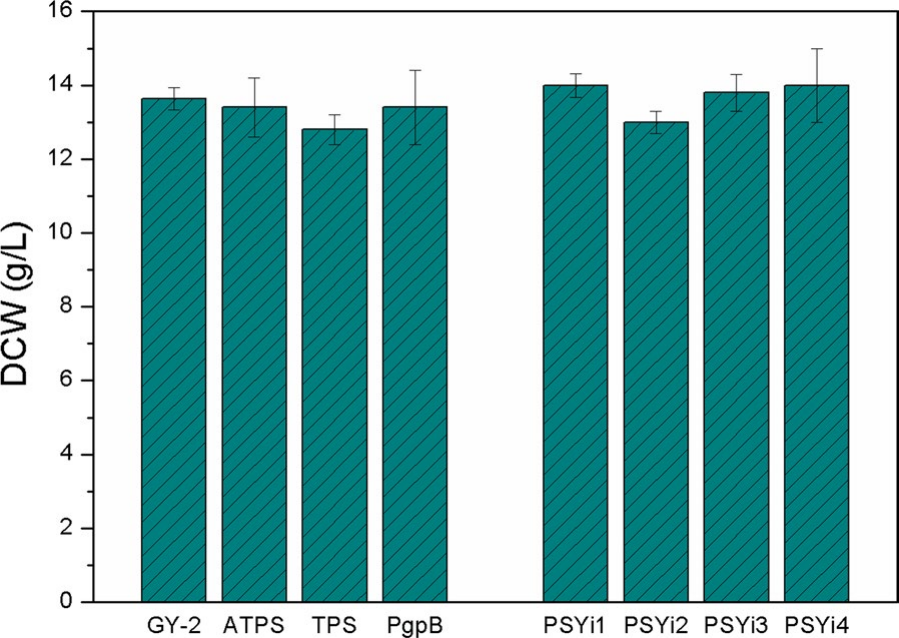
Effects of overexpression of the three exogenous genes and RNA interference of *psy* on the biomass accumulation of *R. sphaeroides* strains. *R. sphaeroides* GY-2 was used as control

**Fig. 4:**
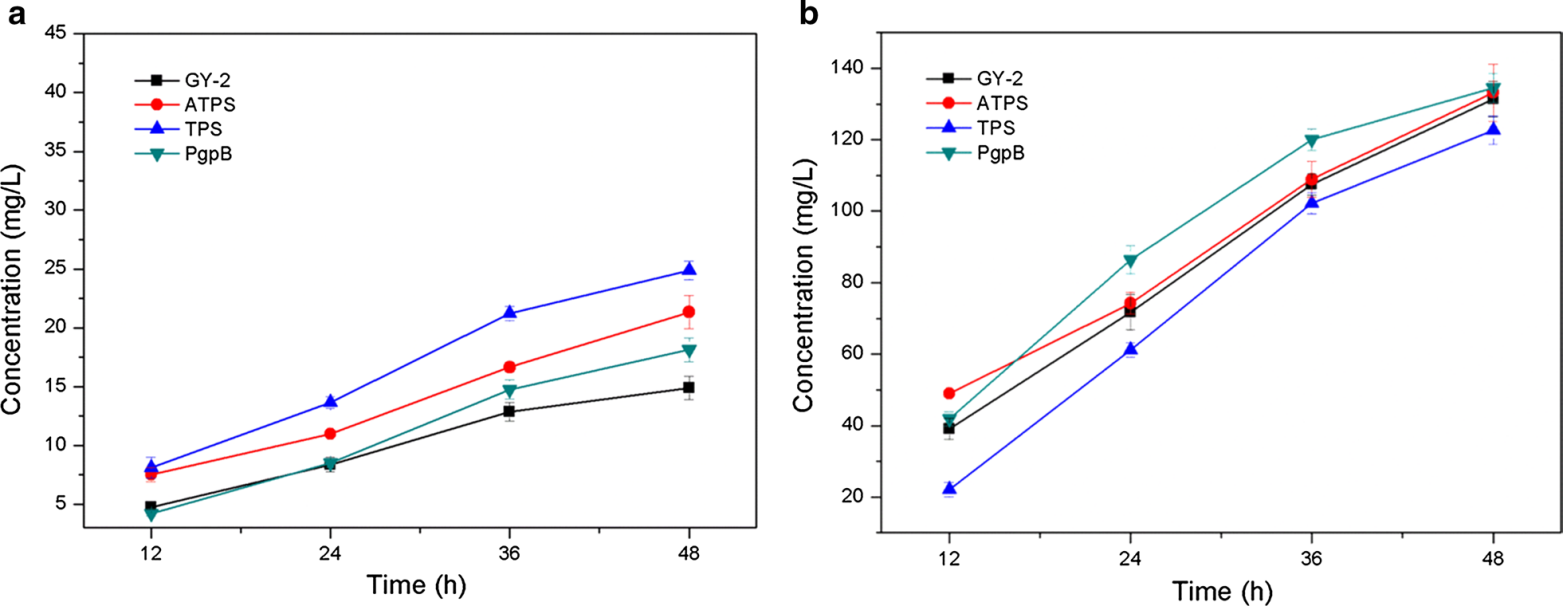
Effects of overexpression of the three exogenous genes on **a** FOH production and **b** CoQ_10_ production in a 48 h of batch culture

FOH can be directly synthesized via FPP dephosphorylation in plants and microorganisms. Phosphatases or pyrophosphatases are suitable for FPP catalysis; however, terpene synthase and sesquiterpene synthase are also expected to be catalyst candidates because FOH biosynthesis usually occurs via terpene synthase or sesquiterpene synthase with broad substrate specificities in plants [1, 33]. In this study, the exogenous terpene synthase (TPS) from *Zea mays* and acyclic sesquiterpene synthase (ATPS) from *Brachypodium distachyon*, as well as the phosphatidylglycerophosphatase B (PgpB) from *E. coli* TOP10 were synthesized, codon optimized for expression, and introduced into *R. sphaeroides* GY-2 for improved FOH production. Figure 3 shows that the introduction of the exogenous genes almost has no effect on the dry cell weight (DCW) of all the strains, except that the biomass accumulation of the *R. sphaeroides* strain harboring TPS was slightly lower than that of the control strain, *R. sphaeroides* GY-2. As we expected, FOH and CoQ_10_ could be co-produced simultaneously from all the metabolically-engineered *R. sphaeroides* strains (Fig. 4). The results showed that FOH production in the three recombinant *R. sphaeroides* strains harboring TPS (Rs-TPS), ATPS (Rs-ATPS), and PgpB (Rs-PgpB) was higher than that of the *R. sphaeroides* strain harboring the plasmid pBBR1MCS2-tac (control) after 48 h of fermentation (Fig. 4a). Thus, it was concluded that FOH could be synthesized using *R. sphaeroides* GY-2 using its endogenous FOH synthase; however, FOH production could be greatly increased by overexpressing exogenous terpene or sesquiterpene synthase. Rs-TPS, Rs-ATPS, and Rs-PgpB respectively produced 68.2%, 43.4%, and 21.9% higher FOH titers than the control strain. Interestingly, the order of the CoQ_10_ yield in the 3 recombinant *R. sphaeroides* strains was exactly opposite to that of FOH production (Fig. 4b). The highest CoQ_10_ production of 134.5 mg/L was obtained from Rs-PgpB, followed by 133.2 mg/L in Rs-ATPS, and 122.7 mg/L in Rs-TPS, which was lower than the control (131.4 mg/L). As illustrated in Fig. 1, the FOH and CoQ_10_ synthesis process was based on competition for FPP. When FOH production increased, the FPP used for CoQ_10_ synthesis correspondingly decreased. Therefore, higher FOH production leads to lower CoQ_10_ titers in all three recombinant *R. sphaeroides* strains.

### Effect of *psy* RNA interference on FOH and CoQ_10_ production

**Fig. 5:**
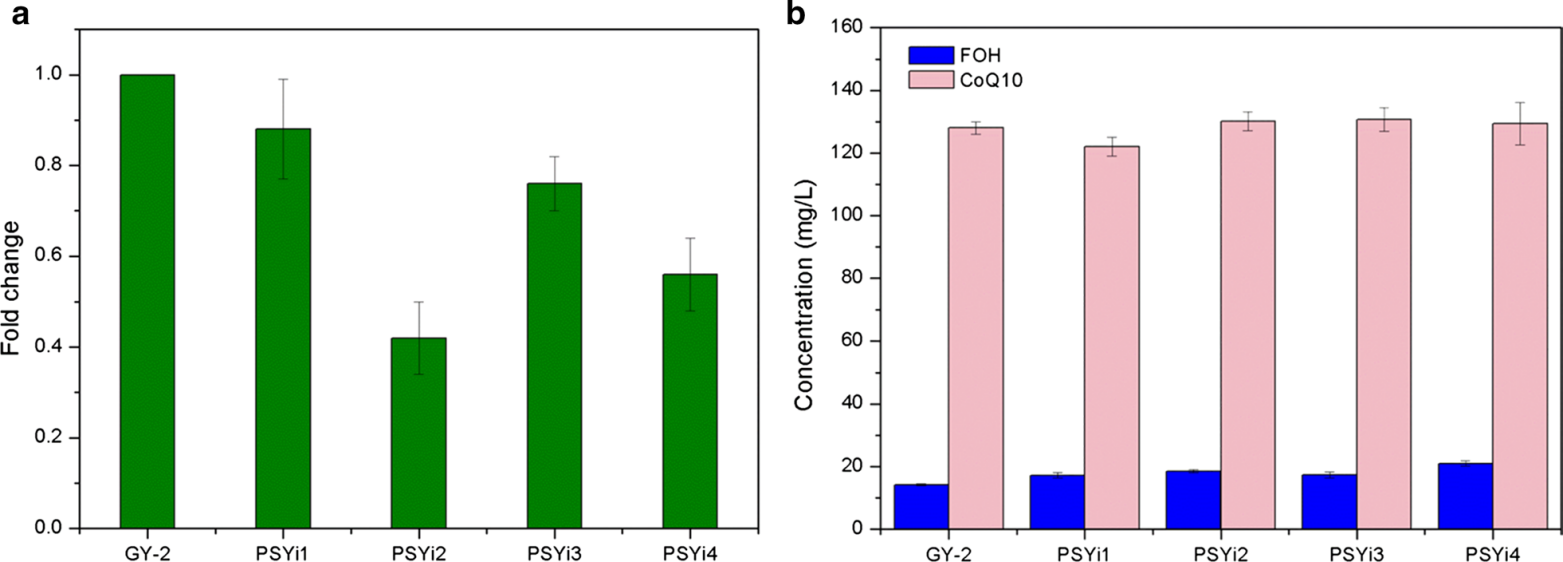
Four *R. sphaeroides* strains with significantly reduced transcriptional levels of gene *psy* were obtained. **a** The different RNA interference levels of *psy* in the four strains, **b** FOH and CoQ_10_ production of the four strains. *R. sphaeroides* GY-2 was used as control

Metabolic flux in microorganisms can be redirected by regulating the key enzymes involved in the pathway. Strategies including overexpression of genes encoding the enzymes for target compound synthesis and disruption of the genes responsible for by-product synthesis or feedback suppression have been developed to improve the titer and yield of the target product [11, 12, 34–36]. Here the plasmid pBBR1MCS2-PSYi was constructed for RNA interference of the gene coding phytoene synthase, which converts geranylgeranyl pyrophosphate (GGPP) molecules into phytoene. pBBR1MCS2-PSYi was obtained and transformed into *R. sphaeroides* GY-2 via bi-parental conjugation. The pre-mRNA was then expressed and spliced to form a self-complementary RNA homologous hairpin comprising of a 365-bp stem and a 62-bp loop by inserting an inverted repeat (IR) sequence. A total of 15 positive *psy* RNA interference transformants were obtained and confirmed via polymerase chain reaction (PCR) and sequencing using the primers, PSYLi-F and PSYSi-R. Among these 15 positive transformants, 4 strains, designated as PSYi1 to PSYi4, showed significantly decreased transcriptional levels of *psy* via a primary screening using quantitative real-time RT-PCR analysis, and were selected as the candidate strains. The biomass accumulation results of these candidate strains illustrated that there was no significant difference in the DCW between the 4 *psy* interference strains and the control *R. sphaeroides* GY-2 strain (Fig. 3). The DCW of the interference strain cultures could reach up to 14 g/L after 48 h, which was only slightly higher than the control. However, the different *psy* RNA interference levels in the 4 candidate strains were obtained based on RNAi-mediated gene silencing via pBBR1MCS2-PSYi (Fig. 5a). The expression level of *psy* in the PSYi2 strain was inhibited by 60%, whereas only 12% inhibition was achieved in PSYi1. This was probably because of the different expression levels of the RNAi or generated siRNA caused due to the different integration sites of the RNAi expression cassette.

FOH and CoQ_10_ production in all the PSY interference strains was determined using GC–MS and HPLC, respectively (Fig. 5b). The results showed that the final FOH production from all the 4 PSY interference strains was significantly higher than that of the control *R. sphaeroides* GY-2 strain. The highest FOH yield of 20.98 mg/L was obtained from PSYi4, while *R. sphaeroides* GY-2 only accumulated 14.2 mg/L, which was 32.3% less than PSYi4. At least more than 20% FOH production was obtained in all the 4 PSY interference strains when compared to the control. The FOH yield from all the PSYi strains was shown to have significantly improved because of the redirection of the carbon flux. The phytoene synthesis pathway was weakened via *psy* RNA interference, which led to the original flux being directed towards phytoene synthesis and eventually, FOH or CoQ_10_ synthesis. However, the PSYi2, PSYi3, and PSYi4 CoQ_10_ titers were not more than 5% higher than the control strain, while the PSYi1 CoQ_10_ yield was lower compared to the control (Fig. 5b). These results demonstrated that CoQ_10_ production in the *R. sphaeroides* strains was almost unaffected by the *psy* RNA interference and redistributed flux, which suggests that most of the redirected carbon flux from the reduced phytoene synthesis likely contributed to FOH production. Due to the high inhibitory effect of the RNA interference, this RNAi-mediated *psy* silencing strategy was utilized to further improve the FOH and CoQ_10_ yields.

### Co-production of FOH and CoQ_10_ from metabolically-engineered *R. sphaeroides*

**Fig. 6:**
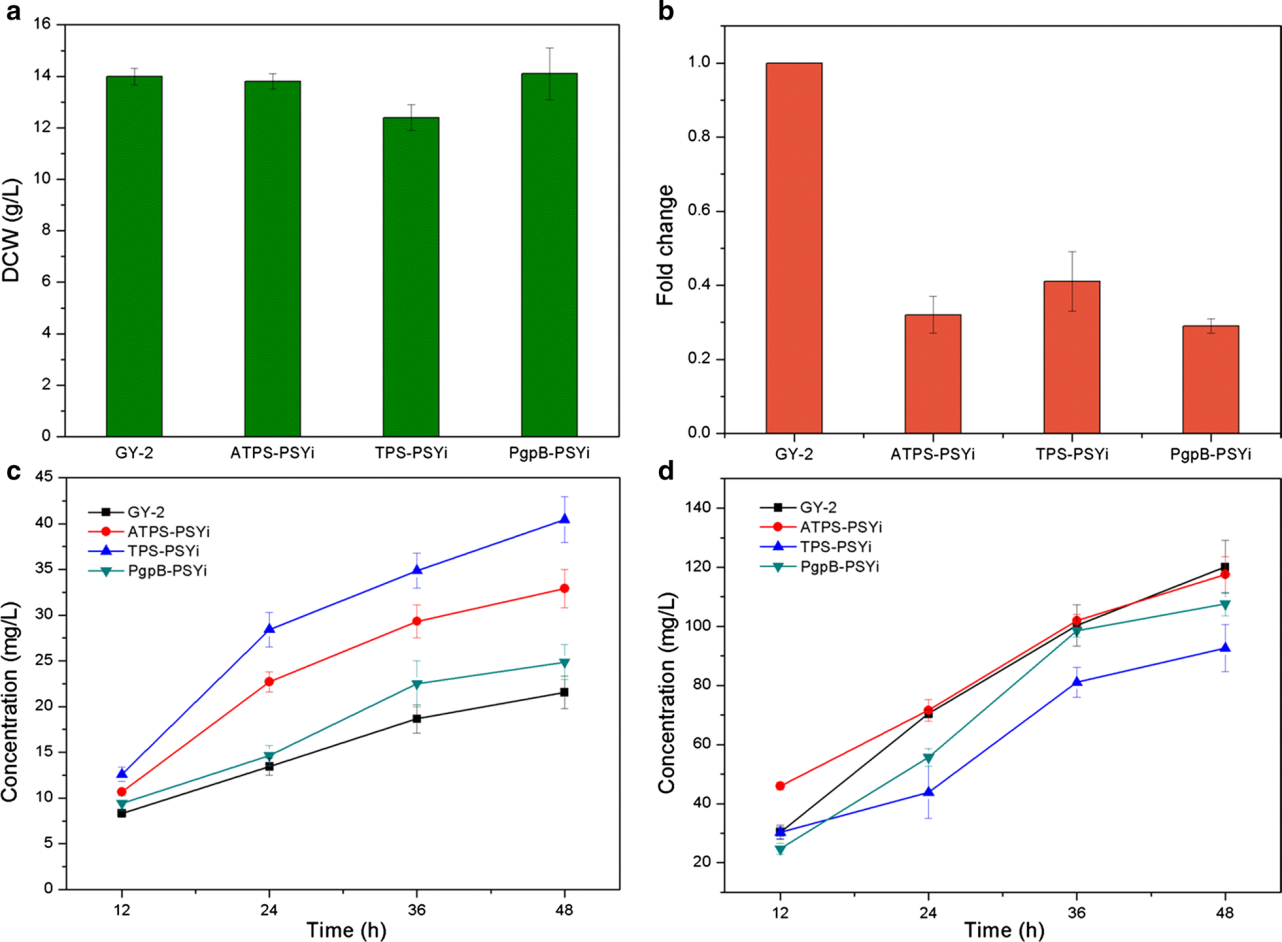
The metabolically engineered strains TPS-PSYi, ATPS-PSYi and PgpB-PSYi were obtained. **a** Biomass accumulation of TPS-PSYi, ATPS-PSYi and PgpB-PSYi. **b** Transcription levels of gene *psy* of these three *R. sphaeroides* strains were differently inhibited. **c** FOH production and **d** CoQ_10_ production of TPS-PSYi, ATPS-PSYi and PgpB-PSYi in a 48 h of batch culture

In the *R. sphaeroides* strains, carbon flux competition at the FPP metabolic junction is distributed into different pathways including lycopene synthesis, ubiquinone pathway for CoQ_10_ production, and squalene biosynthesis. In this study, we opted to reduce the cellular metabolic flux toward squalene biosynthesis in order to enhance the co-production of FOH and CoQ_10_. Thus, the pBBR1MCS2-PSYi RNA interference plasmid was transformed into Rs-TPS, Rs-ATPS, and Rs-PgpB, and the resulting strains were designated as TPS-PSYi, ATPS-PSYi, and PgpB-PSYi, respectively. Similar to the DCW of the recombinant *R. sphaeroides* strains in Fig. 2, the TPS-PSYi biomass accumulation was lower than that of the control strain, while no significant difference was found between the ATPS-PSYi, PgpB-PSYi, and control strains (Fig. 6a). When pBBR1MCS2-PSYi was transformed into Rs-TPS, Rs-ATPS, and Rs-PgpB, *psy* transcription of these 3 *R. sphaeroides* strains was significantly inhibited (Fig. 6b). More than a 50% decrease in *psy* transcriptional inhibition was obtained by RNAi in all the 3 strains. Among the 3 RNAi strains, Rs-PgpB was the most inhibited *R. sphaeroides* strain with only 28.7% transcription remaining when compared to the control. Theoretically, a higher flux through FPP redirects into the ubiquinone and FOH synthesis pathway. Figure 6c demonstrates the FOH production from the metabolically-engineered strains, TPS-PSYi, ATPS-PSYi, and PgpB-PSYi, within 48 h of fermentation. The highest FOH production of 40.45 mg/L was obtained from TPS-PSYi in a batch culture of 48 h, which was twice as high as that of the control strain. Moreover, FOH production from ATPS-PSYi and PgpB-PSYi were 52.8% and 15.6% higher than that of the control, respectively. These results indicate that FOH production can be substantially increased by overexpressing terpene or sesquiterpene synthase coupled with the weakening of the phytoene synthesis pathway via *psy* RNA interference. Although the highest efficacy of RNA interference was found in PgpB-PSYi, the lowest FOH production was also observed in this strain; this is different from the PSYi2 results, where it was the most inhibited strain but exhibited higher FOH production (Fig. 5). CoQ_10_ production in TPS-PSYi, ATPS-PSYi and PgpB-PSYi was no more than 120 mg/L, which was lower than that of the control strain, *R. sphaeroides* GY-2 (Fig. 6d). ATPS-PSYi and TPS-PSYi accumulated the highest and the lowest CoQ_10_ titers, respectively. CoQ_10_ production in TPS-PSYi, ATPS-PSYi, and PgpB-PSYi was found to be significantly and respectively lower than the Rs-TPS, Rs-ATPS, and Rs-PgpB overexpression strains. However, no corresponding relationship for the redirecting carbon flux was observed because both FOH and CoQ_10_ production in ATPS-PSYi was higher than that in PgpB-PSYi.

## Discussion

Farnesol, an acyclic sesquiterpene alcohol, is found in the essential oils of various plants in nature and is used to treat allergic asthma, gliosis, diabetes, atherosclerosis, and hyperlipidemia [8]. Exogenous FOH has been reported to regulate inflammatory responses and exhibit anti-cancerous and anti-inflammatory effects [8, 9]. FOH, extracted from natural plants, has been used in chemoprevention due to its relatively low toxicity as compared to its synthetic counterparts; however, its extraction cannot meet large scale needs because limited amounts of FOH content in plant oil leads to high extraction costs. There is a growing interest in engineering terpene and sesquiterpene production for large-scale fermentation using microorganisms to circumvent the limited availability of these valuable compounds with low natural content [12]. In this study, we firstly produced FOH from a metabolically-engineered *R. sphaeroides* mutant strain GY-2 that already provides high CoQ_10_ yields, which is also a valued compound with many clinical benefits [16]. FOH and CoQ_10_ can be co-produced simultaneously and separated easily from one another because the synthesized FOH is secreted outside cells, whereas CoQ_10_ is produced and maintained inside the cells. Although FOH can be synthesized from *R. sphaeroides* GY-2, it is almost impossible to obtain more than 15 mg/L of FOH due to the low enzymatic activities of endogenous FOH synthase or pyrophosphorylase in the control strain. FOH production was significantly increased by overexpressing PgpB from *Escherichia coli*, TPS from *Zea mays*, and ATPS from *Brachypodium distachyon* in *R. sphaeroides* GY-2. However, CoQ_10_ production decreased correspondingly due to the enhanced carbon flux directed toward FOH synthesis. These results suggested that an improved flux, including the portion originally used for CoQ_10_ production, was redirected toward FOH synthesis through metabolic modification.

On the other hand, the reaction catalyzed by PSY, which was the first committed step of the carotenoid synthesis, was considered to be an important control point in regulating the carbon flux through the carotenoid synthesis pathway [37]. Carotenoids in microorganisms are considered to be biological antioxidants against reactive oxygen species that relieve the damage caused by oxidative DNA-damaging agents [38]. RNA interference mediated by the expression of short hairpin RNAs (shRNAs) is a powerful tool in efficiently suppressing target genes. Thus, RNA interference-mediated silencing, rather than disruption, of *psy* was carried out in this work to weaken the phytoene synthesis pathway so that the carbon flux directed towards FOH and CoQ_10_ could be further improved. Phytoene production was decreased by 26.3% when compared to the control in the PSY interference strains (Additional file 1: Fig. S1), resulting in improved FOH production.

There is probably not much flux being directed into the functional carotenogenic pathway because only a small amount of carotenoid can be found in many organisms [39]. However, improved FOH production was obtained in all four PSY interference strains when compared to the control strain, whereas CoQ_10_ production in these strains was almost unaffected (Fig. 5). In fact, conditional inhibition of the specific pathway after sufficient bacterial growth has been shown to redirect the excess flux toward a desired bio-production pathway [40]. The results suggested that most of the redirected excess carbon flux caused by the interruption of phytoene synthesis likely contributed to FOH production. An alternative explanation to the results is that the flux redirected towards improved FOH production was far more significant than that toward CoQ_10_ production because the same amount of redirected flux from the reduced phytoene synthesis contributed to almost 4 times more FOH than CoQ_10_ due to the different carbon contents of their molecules. Furthermore, FOH production was greatly improved by the three metabolically-engineered strains combined with the terpene or sesquiterpene synthase overexpression and *psy* RNA interference (Fig. 6c). CoQ_10_ production in these strains was also significantly decreased and even lower than that of the control strain, *R. sphaeroides* GY-2 (Fig. 6d). This confirmed the previous suggestion that the improved flux that was originally involved in CoQ_10_ production was being redirected towards FOH synthesis through metabolic modification. It appears that the carbon flux balance can be disturbed and redirected when a related metabolic pathway changes. In this study, a generated carbon flux derived from multiple sources at the FPP metabolic junction was redistributed towards FOH synthesis by introducing exogenous terpene or sesquiterpene synthase. The TPS-PSYi and ATPS-PSYi strains exhibited potential industrial application prospects due to their ability to simultaneously co-produce high levels of FOH and CoQ_10_. However, the highest FOH titer of 40.45 mg/L obtained from TPS-PSYi in this study was still lower than the previous reports [10, 11] due to lack of MVA pathway in *R. sphaeroides*. Despite this disadvantage, the strategy in this work for effective metabolic flux redirection is convenient and economical for the possible commercial co-production of FOH and CoQ_10_. Further improvements in FOH and CoQ_10_ production can be possibly accomplished through metabolic engineering for more available carbon flux redirected towards the desired products.

## Conclusions

In this study, co-production of FOH and CoQ_10_ was performed by introducing three different exogenous terpenes or sesquiterpene synthases combined with *psy* RNA interference in *R. sphaeroides* GY-2. It is noteworthy that FOH production was greatly enhanced due to redirected flux derived from the carotenoid and CoQ_10_ synthesis pathways. This study confirms the effectiveness of *psy* RNA interference in redirecting carbon flux, responsible for phytoene synthesis, towards FOH production. To the best of our knowledge, this is the first reported instance of simultaneous FOH and CoQ_10_ co-production in *R. sphaeroides* using the metabolic engineering strategy. This strategy can broaden applications in other organisms for further improvements in desired products. Therefore, the two strains, TPS-PSYi and ATPS-PSYi, developed in this study provide a viable approach using strain engineering for the industrial production of FOH and CoQ_10_.

## Additional file

**Additional file 1: Fig. S1.** Phytoene production of the four strains with RNAi mediated silencing of the gene *psy*. *R. sphaeroides* GY-2 was used as the control.

## Data Availability

The datasets used and/or analyzed during the current study are included in this article and available from the corresponding author on reasonable request.

## Abbreviations

PSY: phytoene synthase
PPFPP: prephytoene diphosphate
GGPP: geranylgeranyl-PP
UbiA: 4HBA-polyprenyltransferase
UbiD, B, E, F, G, H: quinoid ring modification enzymes
DMAPP: dimethylallyl-PP
IPP: isopentenyl-PP
GPP: geranyl-PP
FPP: (E,E)-farnesyl-PP
GGPP: geranylgeranyl-PP
IDI: isopentenyl-diphosphate delta-isomerase
DXR: 1-deoxy-d-xylulose-5-phosphate reductoisomerase
ispD: 4-diphosphocytidyl-2-C-methyl-d-erythritol synthase (IspD)
ispE: 4-diphosphocytidyl-2-C-methyl-d-erythritol kinase (IspE)
ispF: 2-C-methyl-d-erythritol-2,4-cyclodiphosphate synthase (IspF)
ispG: 4-hydroxy-3-methyl-but-2-enyl pyrophosphate synthase (IspG)
ispH: 4-hydroxy-3-methyl- but-2-enyl pyrophosphate reductase (IspH)
MvaE: bifunctional acetoacetyl-CoA thiolase and HMG-CoA reductase
MvaS: hMG-CoA synthase
MvaK1: mevalonate kinase
MvaK2: phosphomevalonate kinase
HMG-CoA: hydroxymethylglutaryl-CoA
MVA: mevalonate
MVA-P: mevalonate 5-phosphate
MVA-PP: mevalonate pyrophosphate
