# Supplementary figures and images for: Co-production of farnesol and coenzyme Q10 from metabolically engineered Rhodobacter sphaeroides
Source: Microb Cell Fact. 2019 May 31;18:98. doi: 10.1186/s12934-019-1145-6 (PMC6544981; doi:10.1186/s12934-019-1145-6)

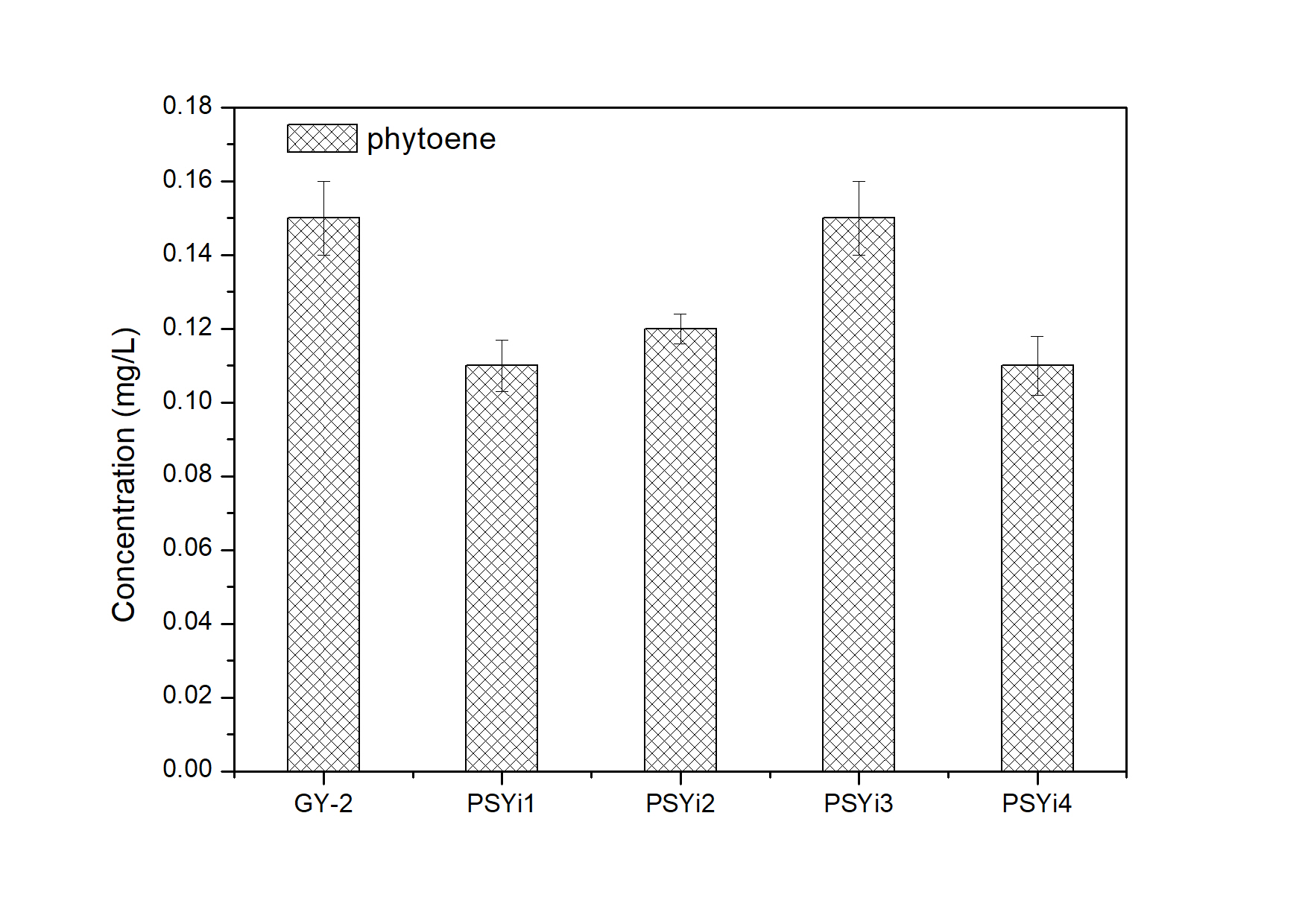

Supplement: Supplementary file 1 — Additional file 1: Fig. S1. Phytoene production of the four strains with RNAi mediated silencing of the gene psy. R. sphaeroides GY-2 was used as the control. [file 12934_2019_1145_MOESM1_ESM.jpg]
